# Amygdala Circuitry During Neurofeedback Training and Symptoms’ Change in Adolescents With Varying Depression

**DOI:** 10.3389/fnbeh.2020.00110

**Published:** 2020-07-22

**Authors:** Karina Quevedo, Jia Yuan Teoh, Maggie Engstrom, Riley Wedan, Carmen Santana-Gonzalez, Betanya Zewde, David Porter, Kathrin Cohen Kadosh

**Affiliations:** ^1^Department of Psychiatry, Medical School, University of Minnesota, Minneapolis, MN, United States; ^2^Minnesota Supercomputing Institute, University of Minnesota, Minneapolis, MN, United States; ^3^School of Psychology, University of Surrey, Guildford, United Kingdom

**Keywords:** amygdala circuits, neurofeedback, emotion regulation, depression, adolescence, prefrontal cortex, rumination

## Abstract

Typical adolescents have increased limbic engagement unchecked by regulatory medial prefrontal cortex (PFC) activity as well as heightened self-focus. The resulting emotion dysregulation and self-focused rumination make adolescents more susceptible to depression and suicide attempts. Heightened self-focus converges with mental illness among depressed adolescents, who deploy exaggerated attention to negative self-relevant stimuli and neglect positive ones as part of depression’s phenomenology. This results in rigid negative self-representations during an identity formative period with potential lifetime repercussions. Current empirically supported treatments fail to allay recurrent depression. Evidence-based interventions for illnesses linked to suicide ideation and attempts (e.g., depression) underperform across the lifespan. This could be because current treatments are not successful in altering pervasive negative self-representations and affect dysregulation, which is known to be a risk factor of chronic depression. This study departs from the premise that increasing positive self-processing might be protective against chronic depression particularly during adolescence. The present research is a novel investigation of neurofeedback as a potential treatment alternative for adolescent depression. To enhance positive self-processing, we used the happy self-face as a cue to initiate neurofeedback from the bilateral amygdala and hippocampus and adolescents attempted to upregulate that limbic activity through the recall of positive autobiographical memories. We identified limbic functional circuitry engaged during neurofeedback and links to short-term symptoms’ change in depression and rumination. We found that depressed youth showed greater right amygdala to right frontocortical connectivity and lower left amygdala to right frontocortical connectivity compared to healthy controls during neurofeedback vs. control conditions. Depressed youth also showed significant symptom reduction. Connectivity between the right amygdala and frontocortical regions was positively correlated with rumination and depression change, but connectivity between frontocortical regions and the left amygdala was negatively correlated with depression change. The results suggest that depressed youth might engage implicit emotion regulation circuitry while healthy youth recruit explicit emotion regulation circuits during neurofeedback. Our findings support a compensatory approach (i.e., target the right amygdala) during future neurofeedback interventions in depressed youth. Future work ought to include a placebo condition or group.

## Introduction

Adolescence is a period of increased risk for developing depression, and early onset is associated with a poorer prognosis, higher symptom severity, and comorbidity, along with higher suicidality rates (Sung et al., 2013). Given that suicide—a severe outcome of depression in youth—is the second leading cause of adolescent death in the United States (Murphy et al., 2018) and that persistent depression is a risk for adolescent attempts (Goldstein et al., 2012), the need for developing novel and effective treatments for adolescent depression is imperative. This is particularly critical as it has been shown that whereas current treatments such as medication and psychotherapy aid in recovering from depressive episodes, they often fail to prevent recurring episodes (Rush et al., 2008; Maalouf and Brent, 2012; Schwartz-Lifshitz et al., 2012; De Silva et al., 2013; O’Connor et al., 2013; Hetrick et al., 2016; Weisz et al., 2017; Cha et al., 2018), resulting in a compounded economic burden for families and health systems (Wang et al., 2003) and higher risks for suicide attempts (Pezawas et al., 2003; Witte et al., 2009; Jonsson et al., 2011). This is a pre-clinical study comparing healthy and depressed adolescents’ functional connectivity (FC) during a novel neurofeedback task that upregulated the activity of limbic sub-cortical and temporal regions of interest (ROI: bilateral amygdala and hippocampus) with the expectation that modulating their activity would cascade to beneficial connectivity changes that are the focus of the present follow-up publication. The publication of the ROI activity results (Quevedo et al., 2019) precedes the current report which follows-up that work and extends its significance for short-term symptoms change and connectivity patterns.

Adolescence is characterized by heightened engagement of emotional subcortical limbic systems such as the amygdala, yet regulatory medial prefrontal cortical (mPFC) systems remain relatively immature (Arruda-Carvalho et al., 2017). This leads to strong emotional experiences among typically developing youth which is unchecked by regulatory control by the mPFC (Hare et al., 2008). Typical adolescent brain development might, therefore, contribute to the onset and severity of depression due to difficulty experiencing and sustaining positive emotions (that along with exacerbated negative emotions characterizes depression) converging with immature emotion regulation and self-identity (Ahmed et al., 2015). In addition to developing brain circuitry of emotion and its regulation, adolescence is characterized by self-processing transformations due to maturing cortical neural circuitry. Self-processing involves conscious and unconscious processes of relating external information to one’s self, such as self-recognition in photographs or self-attribution of traits and skills (Nejad et al., 2013). Conversely, due to heightened cortico-limbic plasticity (Gee et al., 2013), this developmental period is a window of opportunity to train the substrates of affect regulation and self-processing *via* novel procedures such as neurofeedback.

Neurofeedback utilizes the latest developments of real-time functional magnetic resonance imaging (rt-fMRI) data processing and pattern analysis to train participants in the self-modulation of neural networks (Johnston et al., 2010). Neurofeedback allows for localization and voluntary modulation of brain activity “*in vivo*” (Marzbani et al., 2016) as individuals visualize neural activity levels from ROIs inside the scanner. The strength of this technique lies in its high spatial resolution, which allows us to probe functionally connected networks, including deep subcortical structures (Paret et al., 2019). Neurofeedback combines neuromodulation and emotion regulation (Linhartová et al., 2019). It is well suited to non-invasively study dynamic self-processing and affect regulation in youth. Our goals were to: (1) engage cortico-limbic circuits by increasing bilateral amygdala and hippocampus activity; (2) compare depressed and healthy adolescents during neurofeedback targeting cortico-limbic circuits of positive self-processing; and (3) garner preliminary neural data linked to depression and rumination short-term changes in depressed youth.

Previous research has successfully used neurofeedback in psychiatric patients. For example, Young and colleagues used autobiographical memory recall during neurofeedback to enhance activity in the amygdala (Young et al., 2014, 2017a,b, 2018). Healthy adults show enhanced amygdala-mPFC connectivity during autobiographical memory recall during neurofeedback (Zotev et al., 2013). Depressed adults showed a reversal of hypoactive connectivity between the amygdala and cortical areas before vs. after neurofeedback training during resting state conditions (Yuan et al., 2014). Specifically, they showed strengthened amygdala connectivity with frontotemporal networks, such as the precuneus and mPFC components, prominently the rostral anterior cingulate cortex (rACC), after neurofeedback training (Yuan et al., 2014). In summary, the neurofeedback literature in depressed adults shows that corticolimbic FC is strengthened in depressed adults after neurofeedback that targets single ROI activity.

Multiple neurofeedback studies of psychiatric illnesses have modulated single ROI’s activity, yet symptom improvements occurred *via* ROI connectivity changes (Yuan et al., 2014; Paret et al., 2016; Young et al., 2018). One such study found that real-time functional magnetic resonance imaging (rt-fMRI) neurofeedback targeting the amygdala’s activity increased amygdala-lateral prefrontal cortex (PFC) connectivity in borderline personality disorder patients (Paret et al., 2016). Critically, increases in connectivity were correlated with decreased emotional awareness and dissociation, indicating a relief in emotion regulation and self-processing symptoms (Paret et al., 2016). Additionally, the upregulation of the left amygdala activity *via* rt-fMRI neurofeedback led to increased amygdala connectivity post vs. pre-neurofeedback during resting state conditions (Yuan et al., 2014). Similarly, targeting the left amygdala with rt-fMRI neurofeedback resulted in higher amygdala connectivity with frontal and limbic structures (Young et al., 2018). Overall, prior neurofeedback research has therefore shown that changes in connectivity are associated with decreased depressive symptoms and increased ability to recall specific memories (Young et al., 2017b). Finally, the downregulation of amygdala activity *via* neurofeedback in post-traumatic stress disorder (PTSD) patients resulted in decreased symptom severity and increased connectivity between the amygdala and the dorsolateral and dorsomedial prefrontal (Nicholson et al., 2017, 2018). To summarize, multiple studies suggest that single ROI modulation *via* rt-fMRI likely exerts its beneficial effects *via* changes in patterns of ROI-cortical connectivity. Accordingly, we expected that similar amygdala or hippocampus to cortical connectivity could underpin symptoms improvements in the present study. Furthermore, this study’s use of an explicit “top-down” mental strategy was to modulate limbic sub-cortical structures in adolescents by engaging regulatory “top” medial prefrontal cortical (mPFC) systems that are still maturing (Arruda-Carvalho et al., 2017). The goal of our connectivity analyses thus was to test the potential re-routing of cortico-limbic pathways in depressed youth due to the voluntary enhancement of positive stimuli amygdala or hippocampus connectivity.

Limited neurofeedback adolescent trials have found improved symptoms *via* increased inferior PFC activity in healthy adolescents and upregulated emotion regulation networks’ engagement in adolescents with attention deficit hyperactivity disorders (ADHD; Cohen Kadosh et al., 2016; Alegria et al., 2017). FC analyses conducted by the same sample employed by Alegria et al. (2017), revealed that the underlying mechanism for symptom improvement may be the FC differences between the PFC and frontostriatal systems and the default mode network (Rubia et al., 2019). Though unrelated to adolescent depression, these are -to date- the only published pediatric neurofeedback studies aside from our study (Quevedo et al., 2019). Those connectivity studies suggested that emotion-regulation based neurofeedback is feasible among adolescents and that it should target circuits of known abnormal connectivity in pediatric depression perhaps aiming to change their hypo-connectivity patterns to improve symptoms.

In the current study, we used real-time functional magnetic resonance imaging-based neurofeedback to train adolescent participants in the voluntary regulation of the amygdala and hippocampus activity and expected to engage cortical neural networks that enable self-processing and affect regulation. Our selection of ROI was spurred by our findings of the *hypoactive* bilateral amygdala and hippocampus (AMYHIPP) during happy self-face vs. other-face recognition (Quevedo et al., 2018) in depressed adolescents compared to healthy controls. Our decision to examine left and right amygdala connectivity in the present study was also guided by the fact that left vs. right amygdala to mPFC connectivity characterized suicide attempts in depressed adolescents (Alarcón et al., 2019). Specifically, attempting youth engaged left the amygdala-ACC connectivity to a greater degree than all other youth during self-vs. other face recognition (Alarcón et al., 2019). These results suggested that the amygdala hemisphere of connectivity may constitute a critical marker to examine during neurofeedback. Given that our work showed amygdala and hippocampus *hypoactivity* during positive self-relevant information (i.e., happy self-faces), as well altered amygdala-ACC connectivity in attempting depressed youth, we administered a neurofeedback task using the happy self-face as an emotional cue to initiate AMYHIPP neurofeedback and used happy other-face during the control condition. Our activity analyses of that task, in a prior publication, yielded higher AMYHIPP and frontotemporal activity during neurofeedback vs. control conditions and significant modulation of AMYHIPP during neurofeedback vs. the control condition (Quevedo et al., 2019).

Abundant research demonstrates dysfunctional AMYHIPP connectivity during self- and emotional processing in depression (Hastings et al., 2004; Tahmasian et al., 2013; Benson et al., 2014; Belzung et al., 2015; Zheng et al., 2017). The AMYHIPP is interconnected with the mPFC to govern emotionally laden memory recall. The AMYHIPP is also reciprocally interconnected with the ventral and dorsal medial PFC, posterior cingulate cortex, and precuneus (i.e., midline cortical structures) to enable emotion regulation and autobiographical memory (Belzung et al., 2015; Doré et al., 2018). For example, AMYHIPP-midline cortical connectivity increases as positive affect or arousal surges during autobiographical recall (de Voogd et al., 2017; Nawa and Ando, 2019). Scientific reviews suggest that dysfunctional emotion regulation networks (that include the AMYHIPP) underlie ruminative brooding during depression: i.e., dwelling on negative autobiographical memory (Nejad et al., 2013). Our conceptual model is that given extensive evidence that depression severely dampens emotional saliency (limbically mediated) and cognitive awareness (mPFC mediated) of positive self-relevant information (Dere et al., 2010; Köhler et al., 2015), neurofeedback could train those processes *via* AMYHIPP upregulation. Given abnormal self-processing circuitry in depressed adolescents (Quevedo et al., 2016, 2018; Alarcón et al., 2019) and the role of AMYHIPP and midline cortical structure dysfunction as substrates of emotion dysregulation; we sought to increase its activity during adolescence: a formative period for self and emotion processing (Greenberg et al., 2004; Murphy et al., 2010).

Our neurofeedback procedure aimed to up-regulate bilateral amygdala and hippocampus activity *via* recall of positive autobiographical memories. The use of explicit cognitive strategies (e.g., recall of positive memories) was expected to engage cortical areas because such strategies entail associative cognition and voluntary “top-down” PFC areas that enable executive control. Accordingly, we sought to test and identify functional circuitry engaged by neurofeedback in depressed and healthy youth and their links to short-term symptoms’ change. A particularly interesting manifestation of both emotion dysregulation and abnormal self-processing is rumination, i.e., dwelling in negative events and autobiographical memories fueled by unrelenting self-focus, a psychological dimension that has been linked to the onset and maintenance of depressive episodes (Nolen-Hoeksema et al., 2008). Given that adolescents’ proneness for self-focus has been associated with unique risks for depressed mood (Chen et al., 1998). We sought to investigate how corticolimbic circuitry during a novel neurofeedback procedure would be associated with short-term changes in self-reported rumination and depression.

**Figure 1:**
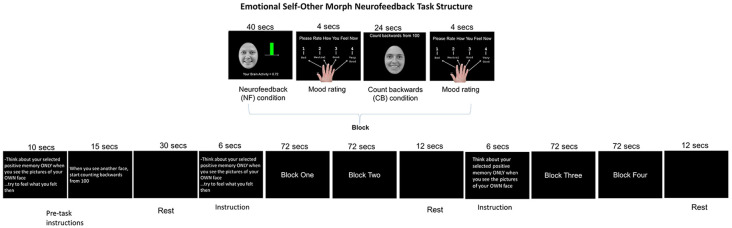
Emotional Self-Other Morph Neurofeedback Task: adolescents recalled happy autobiographical memories during the neurofeedback blocks while seeing their smiling face and counted backward from 100 during the control condition while seeing an unfamiliar face. A shifting colored bar during the neurofeedback blocks was green when the target areas’ activity was above 0 and red for below 0.

The present study follows up on the Quevedo et al. (2019) article which reported activity patterns during and after neurofeedback, but here we report critical connectivity to symptom improvements that indicate possible mechanisms for neurofeedback effectiveness. The goal of the study was to implement a novel neurofeedback protocol in a new population: depressed adolescents. It did not include a placebo group. Specifically, we sought to collect preliminary data for a future full clinical trial. Given the previously reviewed literature on the effects of neurofeedback upon brain connectivity and reported corticolimbic hypoconnectivity in depressed patients and neurofeedback research shows its effectiveness occurs *via* changes in cortical connectivity, we aimed to test the FC of the bilateral amygdala and the hippocampus seeds with PFC areas that support “top-down” emotion regulation and self-processing. The contrast control condition (to compare with the active neurofeedback plus smiling self-face and recall of happy memories) elicited the processing of an unfamiliar adolescent smiling teen face and participants were asked to count backward from one hundred (Figure 1). This condition was selected because it entails both face processing and working memory task that elicit similar brain regions as the active neurofeedback condition. Given cited neurofeedback connectivity we expected that PFC areas associated with self-processing and emotion regulation would be functionally connected to the target amygdala and/or hippocampus seeds during the neurofeedback task. We hypothesized that: (1) all youth would display increased connectivity between the bilateral amygdala or hippocampus seeds and mPFC areas during happy-self face plus feedback (FB) vs. control conditions; and (2) we expected that increased connectivity would be associated to reduced self-reported symptoms before vs. after neurofeedback training. We aimed to increase positive self-processing in depressed youth through facial self-recognition and recall of positive autobiographical memories. The primary objective was to determine the feasibility of using neurofeedback with depressed adolescents to regulate self-processing and emotion regulation circuits and demonstrate their links to self-reported rumination and depression, intending to produce a preliminary data to guide future clinical trials.

## Materials and Methods

Participants were recruited from the community and inpatient units at the University of Minnesota (U of M) using flyers and permission to contact parents. The sample size of 53 participants is significantly larger than the only two pediatric neurofeedback samples published at the time of this work’s submission (Cohen Kadosh et al., 2016; Alegria et al., 2017; Rubia et al., 2019). However, given the study’s novelty with regards to the sample composition and the neurofeedback task, we had no a-priory justification for our sample size. Exclusion criteria included general MRI unsuitability, psychosis, major medical or neurological disorders, and meeting diagnostic criteria for substance abuse or dependance. This study was conducted at the U of M Center for Magnetic Resonance Research and approved by the U of M Institutional Review Board. Right-handed adolescents (*N* = 53, Table 1) with or without depression were evaluated using both categorical (K-SADS-PL, Kaufman et al., 1997) and continuous (CDRS, Poznanski et al., 1979) measures of psychopathology. Author KQ, a licensed clinical child psychologist, established the presence of diagnostic criteria for depression and the absence of past and current psychiatric disorder among control youth. A trained research assistant and KQ examined first sessions’ videos and conferred to establish diagnoses. Pictures of the participants’ faces with a happy, sad, and neutral expression were taken following procedures described in Quevedo et al. (2016). Most depressed youths were on stable medication (Table 1). The present study was not registered because it was not considered a clinical trial at the time of the study’s design and inception, primarily due to the absence of a placebo group.

**Table 1:** Demographics and clinical presentation by diagnostic group.

	Healthy controls	Depressed	Statistic
	*n* = 19	*n* = 34	
Suicide attempters	*n* = 0_a_	*n* = 15_b_
Age at Intake: *M ± SD*	16.26 ± 1.19	16.08 ± 1.27	*F*_(1,51)_ = 0.26
Age: *M ± SD*	16.35 ± 1.23	16.11 ± 1.25	*F*_(1,51)_ = 0.45
IQ: *M ± SD*	115.32 ± 9.12_a_	108.35 ± 10.84_b_	*F*_(1,51)_ = 5.61*
Sex			$\chi_{\left(1\right)}^{2}$ = 0.31
Male	7 (36.84%)	10 (29.41%)	
Female	12 (63.16%)	24 (70.59%)	
Puberty: *M ± SD*	4.53 ± 0.65	4.53 ± 0.68	
Ethnicity			$\chi_{\left(4\right)}^{2}$ = 7.69
White	14 (73.68%)	27 (79.41%)
African American/Black	0	2(5.88%)
American Indian	0	2 (5.88%)
Asian	3 (15.79%)	0	
Other	2 (10.53%)	3 (8.82%)
Family Structure			$\chi_{\left(3\right)}^{2}$ = 2.80
Married	15 (78.95%)	22 (64.71%)
Living with a partner	1 (5.26%)	3 (8.82%)
Separated-Divorced	3 (15.79%)	5 (14.71%)
Single	0	4 (11.76%)
Income			$\chi_{\left(2\right)}^{2}$ = 3.90
≥35K	0	6 (17.65%)
35	#x02013;75K	7 (36.84%)	9 (26.47%)
+>75K	12 (63.16%)	19 (55.88%)
Depression before neurofeedback: *M ± SD*	3.76 ± 3.95_a_	30.5 ± 13.32_b_	*F*_(1,47)_ = 72.01**
Depression after neurofeedback: *M ± SD*	2.26 ± 2.47_a_	21.47 ± 16.33_c_	*F*_(1,51)_ = 25.76**
Rumination before neurofeedback: *M ± SD*	29.31 ± 6.97_a_	50.41 ± 11.77_b_	*F*_(1,47)_ = 49.79**
Rumination after neurofeedback: *M ± SD*	27.89 ± 7.26_a_	44.35 ± 14.35_c_	*F*_(1,51)_ = 21.75**
Depression Severity (CDRS): *M ± SD*	19.21 ± 3.57_a_	49.85 ± 16.15_b_	*F*_(1,51)_ = 66.06**
Depression Diagnosis (K-SADS-PL)
Major Depressive Disorder (MDD)	0	14
MDD with Psychotic Features	0	1
Dysthymia	0	4
Melancholic Depression	0	1
Depressive Disorder-NOS	0	15
Eating Disorders (K-SADS-PL)	0	2	
Anxiety Disorders (K-SADS-PL)	0	22
PTSD (K-SADS-PL)	0	6	
Disruptive Behavior Disorders (K-SADS-PL)	0	6	
Substance Use Presence (K-SADS-PL)	0	2	
Medication
Antidepressants	0	26	$\chi_{\left(1\right)}^{2}$ = 27.53**
Antipsychotics	0	2	$\chi_{\left(1\right)}^{2}$ = 1.10
Mood stabilizers	0	0	
Anxiolytics	0	10	$\chi_{\left(1\right)}^{2}$ = 6.56*

*Note 1: Different letter subscripts (a, b, or c) denotes significant statistical differences between groups or within groups across times of symptoms’ sampling. M, Mean; SD, Standard Deviation; NOS, Not otherwise specified*. *p < 0.05, **p < 0.005, ***p < 0.001. Bold values represent significant difference in *p*-value.

During the first sessions, IQ was sampled (WASI, Weschsler, 1999). During the second session, and before scanning, participants identified and wrote 5–6 positive memories and recalled them during NF blocks. Experimenters helped them to identify peak positive arousal moments within complex memories. Participants completed the Emotional Self-Other Morph Neurofeedback task (ESOM-NF) in the scanner as part of a larger imaging study. Rumination (Treynor et al., 2003), as well as depression questionnaires (Angold et al., 1995; Messer et al., 1995), were administered before (time 1) and after (time 2) the scanning session. Youth were told to think about the present day. To calculate symptoms change, self-reported symptoms before scanning were subtracted from those reported after scanning [rumination before neurofeedback (T1) minus rumination after neurofeedback (T2) = rumination change and depression before neurofeedback (T1) minus depression after neurofeedback (T2) = depression change]. Thus, higher scores indicate lower symptoms after scanning. Finally, participants reported happiness and ease of recall, and those results are reported online in the Supplementary Text II. Correlations between symptoms change and estimates of amygdala FC were conducted across both depressed and healthy participants for the total sample size of (*N* = 53) and are depicted in Figures 3, 4.

**Figure 2:**
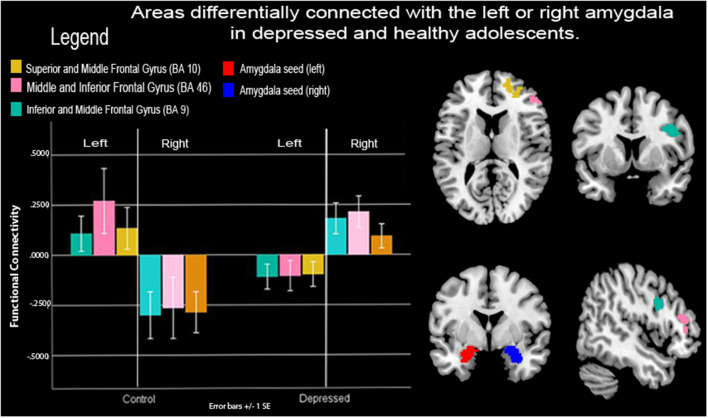
Functional connectivity (FC) between amygdalae and right prefrontal cortex (PFC) during neurofeedback in depressed and healthy adolescents. Depressed adolescents exhibited lower-left vs. right amygdala connectivity to right frontocortical areas during a neurofeedback condition that prompted the recall of positive autobiographical memories cued to a happy-self face image vs. a control condition that prompted counting backward cued to a happy unfamiliar face. Healthy control adolescents showed the opposite pattern. Note: SE, Standard error of the mean.

**Figure 3:**
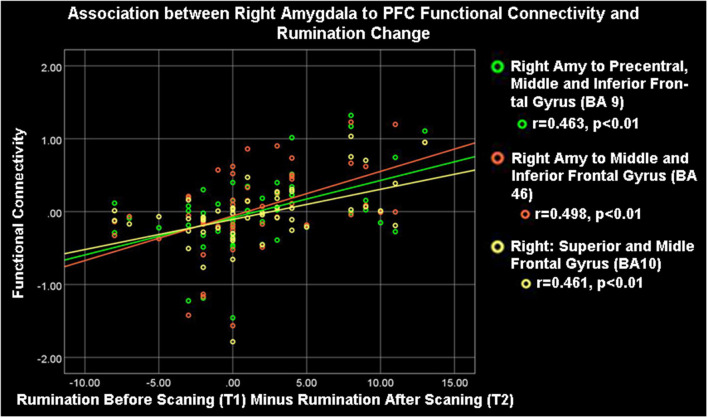
Right amygdala (Amy) to ipsilateral frontocortical connectivity and rumination change. High rumination change was significantly associated with high right amygdala-PFC connectivity during the neurofeedback procedure (ESOM-NF task).

**Figure 4:**
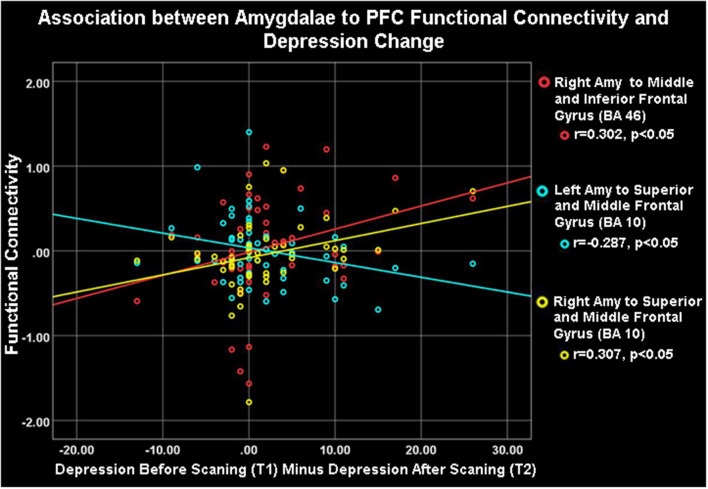
Amygdalae to right frontocortical areas connectivity and depression change. Depression change was positively correlated with right amygdala- ipsilateral PFC connectivity (red and yellow) but negatively associated with left amygdala- right PFC connectivity (blue) during the neurofeedback procedure (ESOM-NF task).

### Neurofeedback Task

#### Emotional Self-Other Morph Neurofeedback, ESOM-NF

This task [duration time (*t*) = 354 s] included four blocks of alternating FB and control (CB) conditions (*t* = 72 s). Blocks were comprised of one FB condition (*t* = 40 s) followed by the participants rating their effect (1 = bad to 4 = good; *t* = 4 s) followed by one CB condition (*t* = 24 s) and finishing with the participants rating their effect again, *t* = 4 s. The FB condition started with participants seeing their happy face (happy self-face) and being asked to increase AMYHIPP activity displayed *via* a colored bar shifting up or down (green = activity > 0, red = activity < 0) brain activity values provided by MURPHI software (Hinds et al., 2011). To influence AMYHIPP activity, participants recalled happy memories discussed before scanning. The CB control condition was cued to an unfamiliar teen happy face (happy other-face) and participants counted backward from 100 with no visual FB. Three rests occurred at beginning (*t* = 30 s, middle (onset = 180 s *t* = 12 s and task’s end (onset = 342 s *t* = 12 s. Participants also viewed instructions for the FB task for 6 s after the first and second rests and instructions for the FB and CB conditions for 25 s before the onset of the first block. Affect rating results have been reported in Quevedo et al. (2019) freely available online.

Both rest and counting-backward conditions have been used as contrasts to test neuromodulation effects in regions of interest (ROI) during neurofeedback protocols. Given the novel nature of our research, as noted in a review about control conditions or groups in neurofeedback designs (Sorger et al., 2019) the lack of a placebo group is acceptable for preliminary proof-of-concept design. However, we chose a control or contrast condition entailing counting backward paired with recognizing an unfamiliar face. It was expected that this would engage areas supportive of both working memories during the counting-backward condition (Woodward et al., 2006) and face processing during exposure to an unfamiliar teen face (Sabatinelli et al., 2011). It was expected that both the FB and CB conditions would engage circuits supportive of memory recall (Quevedo et al., 2019) and self-face and unfamiliar processing (Quevedo et al., 2016, 2018; Alarcón et al., 2019) during the novel neurofeedback protocol.

#### Magnetic Resonance Imaging (MRI) Data Acquisition and Processing

Neuroimaging data were collected using a 3.0 Tesla Siemens Prisma MRI scanner with the 32 Channel receive-only head coil. Structural 3D axial MPRAGE images were acquired for each participant (TR/TE: 2,100 ms/3.65 ms; TI: 1,100; Flip Angle 7°; Field of View: 256 × 256 mm; Slice-Thickness: 1 mm; Matrix: 256 × 256; 224 continuous slices), GRAPPA 2. Mean BOLD images were then acquired with a slice-accelerated gradient-echo EPI sequence during 6.08 min for the ESOM_Pre and Post tasks (2.4 mm^3^ voxels, covering 60 oblique axial slices; TR/TE = 1510/32.4 ms; FOV = 216 × 216 mm; matrix 90 × 90; Flip Angle 65°; multi-band acceleration factor 3). Multiband has previously been successfully used for neurofeedback by us and others (Quevedo et al., 2019).

#### Online Analyses

MURFI software (Hinds et al., 2011) generated and sent AMYHIPP activity values during the FB condition displayed with PsychoPy (Peirce, 2007) during the ESOM_NF task using subject-specific anatomical masks of the bilateral AMYHIPP (see Supplementary Text I). MURFI automatically reduces noise during online analyses. Given the real-time nature of the protocol, MURFI supports certain aspects of preprocessing such as motion correction, polynomial trends removal, artifact detection, and regional smoothing, and smoothing within the region used for FB. The FB signal may still have components of confounds that could have been removed such as slice time correction, susceptibility distortion correction, and other confounds such as compcor, retroicor, and physiological noise such as respiration. The bar representing AMYHIPP values were updated as each new fMRI volume was acquired in a continuous reinforcement schedule. Online subject head motion compensation was accomplished using the Siemens PACE/MoCo system (Thesen et al., 2000). FB automatically stopped if movement exceeded 4–3 mm repeatedly (which occurred in just one participant), but participants could re-initiate the ESOM-NF task. ROIs were localized anatomically during the multi-band echo-planar imaging (EPI) series (target functional reference acquisition, see the Supplementary Text I) for each individual and mapped to an individual’s T1 structural brain data. Data were collected using a 3.0 Tesla Siemens Prisma MRI scanner with the 32 Channel receive-only head coil. Structural 3D axial MPRAGE images were acquired for each participant (TR/TE: 2,100 ms/3.65 ms; TI: 1,100; Flip Angle 7°; Field of View: 256 × 256 mm; Slice-Thickness: 1 mm; Matrix: 256 × 256; 224 continuous slices), GRAPPA 2. Mean blood oxygen level-dependent (BOLD) images were then acquired with a slice-accelerated gradient-echo EPI sequence during 6.08 min for the ESOM-Pre and Post tasks and 6.02 min for the ESOM-NF task (2.4 mm^3^ voxels, covering 60 oblique axial slices; TR/TE = 1510/32.4 ms; FOV = 216 × 216 mm; matrix 90 × 90; Flip Angle 65°; multi-band acceleration factor 3). Please see the Supplementary Material for more details of online data processing.

#### Off-Line Data Analysis

SPM12 was used for all fMRI preprocessing and statistical analyses. Preprocessing the EPI time series included: (1) rigid body realignment for head motion correction; (2) slice timing correction; (3) rigid body co-registration of EPI with high-resolution anatomical data; (4) spatial normalization to the Montreal Neurological Institute (MNI) anatomical space using unified segmentation; and (5) spatial smoothing (6 mm full width at half maximum). Head motion outliers in the EPI time series were identified using the Artifact Detection Tools with a scan-to-scan movement threshold of 0.5 mm and a scan-to-scan global signal change of 3 SD^1^. For each subject, the BOLD-contrast signal variance was modeled with a set of regressors using a general linear model. The total signal variance was decomposed into a task component, with inter-trial intervals as implicit baselines. Each task regressor was constructed by generating condition duration vectors and then convolving them with a canonical hemodynamic response function, allowing parameter estimates proportional to task-related neural activity per second. The full model for each subject comprised: (1) the condition regressors; (2) regressors modeling movement-related signal modulation; (3) outlier time points; (4) the mean signal for the session; and (5) a discrete cosine transform basis set that modeled the low frequency, presumably artifactual, signal modulations at frequencies lower than 0.008 Hz. Parameter estimates were calculated using a restricted maximum likelihood algorithm. All offline and online activity analyses have been previously reported by Quevedo et al. (2019).

#### Region of Interest Seed Definitions

FC was determined using psychophysiological interactions (PPI) to explain activity changes in the target ROIs due to an interaction between the activity in the amygdalae and FB+self-face vs. count-backwards+other-face, which served as the contrast or control condition (Friston et al., 1997) during the ESOM_NF task. The seed regions for connectivity analyses were 8 mm diameter spheres placed in the left and right amygdala, as defined by the PickAtlas toolbox (Maldjian et al., 2003). The signal time course in the amygdalae was obtained for each participant and task condition to construct the 1st level PPI estimate variables. The coordinate of the highest activity within the left or right amygdala were the locations for extracting the signal time courses for the FB vs. the count-backward conditions. Because the coordinate of the highest activity was individualized to each participant, this resulted in slightly different peak coordinates (within the right or left amygdala masks) per participant that reflected their highest peak of amygdalae activity. Note that the peak coordinates of amygdalae activation were statistically similar for the diagnostic groups, *F*_(1,49)_ = 0–0.03, *p* = 0.9) and gender, *F*_(1,49)_ = 0.3–0.17, *p* = 0.86–0.68. Peak coordinates are reported in the supplements online (Supplementary Table S1). Similar procedures were followed for the hippocampi, but no significant areas were connected to these seeds for FB+self-face vs. count-backwards+other-face, nor there was any group by a condition or hemisphere interactions. The hippocampus seed area is henceforth omitted from all results.

#### Functional Connectivity (FC) Analyses

Using the 1st level PPI estimates we tested contributions of the amygdalae activity to areas that differed between groups during the ESOM_NF task. The 1st-level PPI activation maps during ESOM_NF were subjected to a 2nd level general linear models (GLM) analysis with the group as between-subject factors and hemisphere as a within-subject factor. Variables that differed by groups (i.e., IQ) were added as a covariate to the GLM. Significant results show neural areas with high or low coupling with the amygdalae. Clusters and coordinates with statistically significant peak connectivity levels with the seed areas (*p*_unc_ < 0.001) are reported. AFNI (v. 18.2.06) was used to correct for multiple comparisons and determine whole-brain, voxel-wise and cluster-extent thresholds (Cox, 1996) *via* 3dClustSim. The ESOM_NF GLM analysis yielded a voxel-wise threshold (*p*_unc_ < 0.001) and cluster-extent threshold of *k* = 122, *α* = 0.01, and only clusters larger than this threshold are reported. Significant main effects and interactions were interrogated with *post hoc* *t*-tests in SPM12 (*p*_unc_ < 0.001). Each pair-wise comparison was masked with a binary mask of the significant cluster (s) from the principal GLM. Estimates from each significant cluster were extracted using the SPM12 “eigenvariate” function to produce plots depicting the direction of effects. To examine associations between FC and symptom change. Specifically, rumination and depression change associations with FC estimates between the amygdalae and cortical areas using SPSS v.24 software. Please note that neural activity results have been published (Quevedo et al., 2019) and are freely available online. Given the lack of a placebo group, to further underscore the importance of amygdalae-PFC connectivity as a potential mechanism of neurofeedback effectiveness, we conducted additional analyses with ROI’s whose connectivity with the amygdala (for FB vs. count-backward) could be conceivably increased and associated with symptoms change. Using the same 2nd level GLM with the group as between-subject factors and amygdala hemisphere as a within-subject factor we examined connectivity patterns with the bilateral occipital cortex, fusiform and ventral striatum regions of interests. Neither of these ROI’s analyses yielded significant estimates of FC with the amygdala.

## Results

### Group by Amygdala Hemisphere Interaction During Neurofeedback

Frontocortical to amygdalae connectivity varied as a function of the hemisphere between depressed and healthy adolescents. Depressed youth showed greater right amygdala to right frontocortical connectivity compared to healthy adolescents during FB vs. count-backward, however, they showed lower left amygdala to right frontocortical connectivity compared to healthy controls. Specifically, connectivity between the right pre-central, superior, inferior, and middle frontal gyri (BA10, 9, 46) and the amygdala varied depending on the hemisphere of those loci of neurofeedback between the groups (Table 2, Figure 2). There were no increases in connectivity between the PFC and the averaged left and right amygdala seeds, all the results pertained to differences between the left or right amygdala connectivity patterns between the groups.

**Table 2:** Connectivity with the amygdalae during the ESOM_NF task.

Whole-brain analyses results	Voxels	Hemisphere	MNI coordinates			*F*
			*x*	*y*	*z*	
Areas differentially connected with the left or right amygdala in depressed and healthy adolescents.						
Precentral, middle and inferior frontal gyrus, BA 9	122	Right	42	08	30	21.42
Middle and inferior frontal gyrus (dorsolateral prefrontal cortex, dPFC), BA 46	130	Right	46	36	16	17.64
Superior and middle frontal gyrus, BA 10	149	Right	28	44	14	17.49

### Symptom’s Change and Amygdala Circuitry During Neurofeedback

As Table 1 shows, depressed youth, as it would be expected, reported higher rumination and depression compared to controls at both time points, yet only depressed youth -again as expected and likely due floor effects in controls—reported significant symptom reduction after scanning procedures (see Table 1). Correlations between symptoms change and circuitry variables found that connectivity between the right precentral, superior, middle, and inferior frontal gyri (BA10, 9, 46) and the right amygdala were positively correlated with rumination change (Table 3, Figure 3) across the whole sample (*N* = 53). Connectivity between the right superior, middle, and inferior frontal gyrus (BA46, 10) and the right amygdala seed was positively correlated with depression change, however connectivity between the superior and middle frontal gyrus (BA10) and the left amygdala seed was negatively correlated with depression change (Table 3, Figure 4).

**Table 3:** Correlations between amygdalae functional connectivity to PFC and depression or rumination change.

		Pearson correlation	
Areas of connectivity with the amygdalae during the ESOM-NF task	Amygdala hemisphere	Rumination change	Depression change
Right Precentral, Middle and Inferior Frontal Gyrus (BA 9)	Left	0.126	−0.192
	Right	0.463**	0.216
Right Middle and Inferior Frontal Gyrus (BA 46)	Left	−0.060	−0.183
	Right	0.498**	0.302*
Right Superior and Middle Frontal Gyrus (BA 10)	Left	−0.160	−0.287*
	Right	0.461**	0.307*

Differences in rumination or depression scores before vs. after scanning were correlated with left and right amygdala connectivity to right frontocortical regions. Note: ** and *indicate 2-tailed p significance < 0.01 or 0.05 level respectively. Bold values represent significant difference in *p*-value.

## Discussion

This study examined changes in amygdala FC during a neurofeedback procedure (ESOM-NF task) that aimed to increase positive emotions and self-processing by increasing the activity of the bilateral amygdala and hippocampus with the expectation of cascading changes in neurofeedback target to mPFC connectivity. Our hypotheses were partially supported. Contrary to our first hypothesis, there was no increase in connectivity between the averaged right and left amygdala and the mPFC during happy-self FB vs. the control condition (i.e., no effect of ESOM-NF condition across amygdala hemisphere and diagnostic group), nor areas of connectivity with the averaged hippocampus. However FC between the right mPFC and the left or the right amygdala did increase for all youth though this happened differentially for depressed and healthy control for happy self-face plus FB vs. the control condition. Importantly, such group by amygdala hemispheres increases in connectivity were absent for other areas that support visual, hedonic, and face processing; suggesting the hypothesized preeminence of PFC areas that support “top-down” emotion regulation and self-processing. Additionally, as we proposed in our second hypothesis there were associations between amygdala increases in FC with mPFC and changes in short term self-reported symptoms of rumination and depression.

### Amygdala to Frontal Cortex Connectivity in Depressed vs. Healthy Adolescents During Neurofeedback

We found that depressed and healthy adolescents differed in FC between the left and right amygdala and right frontocortical areas during the FB vs. the count-backward condition (Figure 2). Right frontocortical areas and their connectivity with the right amygdalae have been implicated in positive mood and approach behaviors as well as on those dimensions’ disfunction (Phillips et al., 2008). Recent reviews of laterality effects in specific brain structures (including the amygdala and the ventromedial PFC) appear to confirm the hypothesis of a right hemisphere dominance for all components of the emotional system (Gianotti, 2019). Importantly Given that youth were recalling positive autobiographical memories and instructed to “feel what you felt then,” the right-sided laterality of these mPFC areas of connectivity with the amygdalae may represent the limbic to cortical (or vice versa) circuitry that enabled their reported higher positive emotions during FB vs. count-backward blocks (Supplemental Text IV online). However, to elicit those memories and emotions, both depressed and control youth engaged different amygdala to PFC circuits during the neurofeedback task. Specifically, depressed youth engaged right amygdala to ipsilateral mPFC areas during recall of positive autobiographical memories and neurofeedback while control youth engaged left amygdala to contralateral mPFC areas.

Compared to the left amygdala, which is more responsive to conscious processing, explicit emotional responses, and language-dependent processing, the right amygdala is believed to be part of a system of automatic saliency processing and implicit emotional responses (Markowitsch, 1998; Morris et al., 1998; Funayama et al., 2001; Phelps, 2006). The right amygdala is also thought to be responsible for short duration responses, implicit mood regulation as well a negative emotional salience, while the left amygdala is possibly implicated in sustained responses, conscious mood regulation and positive emotional salience (Lane and Nadel, 2000; Baas et al., 2004; Costafreda et al., 2007; Sergerie et al., 2008; Dyck et al., 2011). In the context of these functional models of amygdalae circuitry, depressed youth’s lower engagement of left amygdala-right mPFC connectivity while upregulating positive affect and self-processing suggests both depression’s pathophysiology (i.e., depleted explicit regulatory inputs and/or lower positive saliency) and compensatory mechanisms for such deficits. Specifically, depressed youth engaged ipsilateral right amygdala-mPFC circuits during positive emotion and self-processing up-regulation demands paired to the happy self-face. This suggests that during *voluntary* positive affect and self-processing up-regulation, depressed youth recruited circuitry associated with *involuntary* affect regulation. This might explain why in the amygdala and hippocampus activity time-series analyses we found some indications of less efficient modulation in depressed vs. control youth (Quevedo et al., 2019). Involuntary affect regulation circuitry might be less engaged during voluntary positive affect and self-processing up-regulation. Interestingly, unlike the present results, hippocampi, but not amygdale activity was up-regulated during FB vs. count-backward in all youth (Quevedo et al., 2019) in the activity ROI analyses. By contrast here amygdalae -not hippocampi- functional circuitry responded to the neurofeedback condition and was linked to symptoms’ change. A potential explanation is that while amygdalae activity is equally engaged by emotional self and other faces (leading to no significant differences), its functional circuitry is significantly engaged by voluntary affect and self-processing neuromodulation for the neurofeedback vs. the control conditions which were both cued to smiling faces. Absent hippocampi results might be due to the relatively larger size of those seeds, which might have resulted in higher variability of mechanisms across participants and less convergence on a common circuitry. Alternatively, the hippocampi circuitry might not differ for the contrasting conditions (autobiographical recall and counting backward) given that both entailed working memory and memory retrieval.

The areas of increased connectivity with the right and left amygdalae in depressed and control youth respectively are known to support a plethora of higher-order associative and cognitive functions (Siddiqui et al., 2008), including cognitive control and executive function (Ouerchefani et al., 2019), i.e., the dorsolateral PFC (BA 46), self-referential processing (Meyer and Lieberman, 2018), i.e., the superior and medial PFC, BA 10, and emotion regulation (Rive et al., 2013; Eker et al., 2014; Falquez et al., 2014), i.e., the middle and inferior PFC, BA 9). Pre-central cortex involvement hints at the amygdala to motor coordination during emotional autobiographical recall. In summary, increased right or left amygdala connectivity to the inferior frontal gyrus (two clusters encompassed by inferior portions BA 46 and BA 9) during the FB vs. the count-backward condition, may represent higher cortical coordination with the amygdalae during emotion regulation, cognitive control, and self-processing in all youth during neurofeedback.

Scientific results about depressed adolescent’s neural circuitry of emotion regulation are equivocal. The extant research suggests that they exhibit different patterns compared to depressed adults’ reduced PFC-amygdala coupling during emotion regulation (Ahmed et al., 2015). For example, higher connectivity was reported for a “reduce” emotion condition compared to control youth, while viewing negative images. Similarly, while “reappraising” instances of social rejection, depressed adolescents exhibited higher amygdala and hippocampus connectivity with the right frontal pole compared to controls (Ahmed et al., 2015). Methodological differences, i.e., elicitation of varying emotion regulation strategies and use of different stimuli, are present across those and our study. Notably, prior research pertained to the regulation of negative stimuli while our task entailed the use of positive internally and externally generated stimuli. However, like “reduce or reappraise negative stimuli” we elicited an active strategy “increase positive” which also resulted in higher corticolimbic coupling. Yet, prior adolescent emotion regulation research has not reported hemispheric differences. The only exception is our results of left vs. right amygdala connectivity with PFC structures (ventral and dorsal anterior cingulate cortex) during a self-vs. other face recognition task which distinguished suicide attempting youth from all other adolescents (Alarcón et al., 2019) including depressed youth with intense suicide ideation but no history of attempts. This article extends our prior research and suggests that active emotion regulation strategies engage higher amygdala-PFC circuits in all youth (Quevedo et al., 2019), but it might do so *via* implicit vs. explicit processes (at least during neurofeedback) in depressed vs. control youth. This study did not focus on attempting depressed youth (*N* = 15). Yet, given our findings of similar right amygdala-PFC connectivity in depressed youth with and without attempts (Alarcón et al., 2019), enhancing compensatory processes (borne by high right amygdala-PFC coupling) *via* neurofeedback might be speculated to be a mechanism to assuage both recurrent depression and/or suicide attempts.

There are not enough studies with uniformity in measures to definitively conclude the amygdala laterality for implicit vs. explicit, nor local vs. global processing (Baas et al., 2004). Alternative explanations for the group by amygdala hemisphere interactions entail broader functionalities of the left vs. the right hemisphere. For instance, the right hemisphere thought to be biased towards negative thinking, pessimism, inward withdrawal, and self-reflection while the left hemisphere is thought to be biased towards positive emotion, and decision making (Hecht, 2010)–a.k.a. the valence hypothesis. At a neurochemical level, the left hemisphere is associated with more dopamine demanding processes (Hecht, 2010) and may subsequently reinforce behavioral biases through similar lateralized neuropathways (Aberg et al., 2016). Amygdala lateralization studies do show significantly increased activation of the left amygdala during emotion processing (Baas et al., 2004), particularly the left amygdala is thought to modulate positive emotion (Lane and Nadel, 2000; Costafreda et al., 2007; Dyck et al., 2011). This alternative explanation would suggest that depressed youth may have a less responsive left amygdala circuitry, as evidenced by reduced connectivity of this amygdala hemisphere to cortical brain structures during FB vs. counting backward, due to a prevalence of negative vs. positive emotionality. Our results suggest that a compensatory use of neurofeedback targeting the amygdala ought to select the right amygdala or its connectivity with the PFC as a target of neurofeedback: a circuitry that depressed youth can engage and that is associated with symptoms change.

### Rumination or Depression Change and Amygdala Circuitry

Correlations between FC estimates revealed that improvements in rumination and depression after vs. before neurofeedback training were associated with the right amygdala- right PFC structures connectivity. Ipsilateral right amygdala-PFC coupling was positively correlated with a reduction in both rumination and depression (Tables s 1, 3, Figures 3, 4) suggesting that this might be a tentative mechanism for short-term symptoms improvements. Depression scores measured *via* the mood and feelings questionnaires (MFQ, Messer et al., 1995) indicate illness severity but they also overlap with the presence of maladaptive emotion regulation strategies typical of depressive states (i.e., rumination). Rumination is both a maladaptive emotion regulation strategy and a form of negative self-processing (Nolen-Hoeksema et al., 2008; Nejad et al., 2013). Our findings suggest that neurofeedback might alter coordination between the ipsilateral amygdala and PFC in ways that lead to short-term symptom reduction. Perhaps by increasing positive self-processing and/or positive affect. By contrast, reduced left amygdala to right PFC connectivity was linked to reduced depression (though left-right PFC coupling was uncorrelated with rumination change) suggesting that increasing the harmony between these two loci (i.e., coordinated increases or decreases of activity in the left amygdala and right PFC) might not be as effective to lessen short-term symptoms among depressed youth, given that they were the only ones who reported significant depression and rumination reduction after scanning (Table 1). Given the likely role of the left amygdala in sustained responses, language-driven, and conscious mood regulation (Lane and Nadel, 2000; Baas et al., 2004; Costafreda et al., 2007; Sergerie et al., 2008; Dyck et al., 2011); and the role of the right PFC in emotional processing (the right hemisphere hypothesis) as well as in the processing of negative stimuli (the valence hypothesis; Wyczesany et al., 2018; Gainotti, 2019); negative correlations between right PFC-left amygdala coupling and depression improvement might mean that less reliance on sustained/conscious processing of emotional stimuli during memory recall is linked to short-term depression reduction (something akin to savoring recalled positive memories vs. language-driven positive memory recall). Such speculations regarding mental states must cede to caution, however, due to the small sample size and alternative explanations. An overlapping interpretation (more in line with the right hemisphere hypothesis) might be that explicit mood regulation (left amygdala) or associative (right PFC) emotional recall (borne by left amygdala-right PFC circuits) are either inversely associated to short-term depression reduction or not as strongly linked to both depression and rumination reduction as the engagement of ipsilateral right amygdala-PFC circuits which sustain all components of the emotional system (Gianotti, 2019). Another interpretation might be that reducing left amygdala-right PFC and increasing ipsilateral right amygdala-PFC coupling both contribute to short term depression and rumination improvement. However, while the data might suggest engaging circuitry of implicit emotion regulation (i.e., right amygdala-PFC circuits), the strategy deployed (i.e., recalling positive memories) was an explicit one. Though perhaps the right amygdala-PFC circuitry is engaged to a greater extent in depressed vs. control youth as our results show (Figure 2). It is also possible that short term symptoms change can occur through a variety of mechanisms (correlated with ours) that are not captured by the present analyses which focused only on amygdala connectivity patterns. For higher statistical rigor (i.e., larger sample size) we report associations between right and left amygdala-PFC coupling and symptoms change for the entire sample. However, the fact that -unlike controls- depressed youth report significant symptoms change, added to exploratory correlations between symptoms change and connectivity within diagnostic groups (which showed that circuits to symptoms change associations are significant just for depressed youth), suggest that the results are driven by the depressed youth’s circuits engaged during neurofeedback and their short term symptoms change. The caveat that this may represent a regression toward the mean among depressed and a floor effect for the control youth still apply.

In summary, neurofeedback might reduce rumination, known to be associated to onset and maintenance of depression symptoms (Nolen-Hoeksema et al., 2008) and depression symptoms themselves, *via* engagement of implicit emotion regulation circuits or broad right-sided emotion processing circuits, which are preferentially engaged in depressed youth (Figure 2). Our results suggest the use of compensatory mechanisms for emotion regulation and self-processing among depressed adolescents (i.e., right amygdala and/or PFC circuitry) in future neurofeedback clinical trials. However, to confirm these findings, comparisons of right amygdala vs. left amygdala neurofeedback and use of a placebo group are necessary, as well as longitudinal follow-ups to confirm the enduring or temporary effects of neurofeedback.

## Limitations and Future Directions

There are limitations to consider. PPI analyses have alternative explanations. First, possibly the task condition of neurofeedback modulated the contribution of the amygdalae seeds to PFC areas. However, it is equally possible that the contribution of the amygdalae modulated the PFC regions FC during the neurofeedback vs. the control condition (Friston et al., 1997). Second, due to lack of a placebo condition, it is difficult to discern whether rumination and depression changes represent a regression toward the mean, particularly among depressed youth, or an effect of neurofeedback training. For example, short-term symptoms improvement might be due to the placebo effects of being inside the scanner. Plus, self-reported short-term symptoms’ changes could be due to thinking about positive memories alone, and not to neurofeedback training. Therefore, future studies with a placebo group or condition are needed. Also, this is a cross-sectional study with only short-term effects measured. Future studies ought to include longitudinal studies of adolescents with depression and discern any correlations of symptom reduction or brain activity with reduced lifetime occurrences of depression. There are additional considerations and limitations regarding the mechanisms of symptoms change. In our study, bilateral amygdala and hippocampus activity levels were not significantly associated with symptoms change, instead, the amygdala to PFC connectivity levels during the neurofeedback task appear to be linked to symptoms change. This suggests that FC-based neurofeedback (e.g., actually trying to increase right amygdala-PFC connectivity *via* neurofeedback) may be a more effective and powerful way to exert changes in self-reported symptoms. Given the intrinsic interconnected nature of the human brain, it remains an empirical question whether targeting FC between loci vs. targeting one locus of that same circuit leads to similar, worst, or better outcomes in terms of subject’s ability to control their brain, effect sizes in symptoms change, neural target’s engagement and their relationship. Future research ought to examine these questions which would yield both rich pieces of knowledge about the human brain and departure points for future clinical trials.

## Conclusion

This study is important to the field of clinical neuroscience because it builds empirical knowledge about the adolescent brain during depression and neurofeedback, a potential treatment for treatment-resistant depression and suicide risks. We demonstrated that both depressed and healthy adolescents were able to recruit the amygdala and PFC structures during FB vs. count backward in a neurofeedback procedure. This suggests that executive control of emotional decision-making was prevalent, perhaps entailing both conscious and unconscious modulation of emotional experiences during the recall of positive memories. Increased right amygdala-PFC structures connectivity in depressed youth suggests implicit emotion regulation strategies that lead to symptoms’ improvements, while increased left amygdala-PFC structures connectivity in healthy youth suggest explicit emotion regulation. Our results recommend a compensatory use of neurofeedback and the right amygdala and its FC to the mPFC as important loci and cascading neural targets for future neuromodulation in depressed adolescents. Potential strategies include contrasting online single ROI (e.g., right amygdala) to connectivity (e.g., right amygdala to mPFC) neurofeedback as competing targets to test engagement and therapeutic effectiveness. Alternatively, future work could compare cortical “top” (e.g., ventral or dorsal ACC) vs. limbic “down” (e.g., amygdala) single ROI’s activity, or their top vs. down connectivity. The novelty of neurofeedback as a potential therapeutic tool opens manifold avenues of inquiry including basic research in non-diseased populations which is sorely missing to fully explain how this procedure changes brain function and behavior.

## Data Availability Statement

The datasets generated for this study are available on request to the corresponding author.

## Ethics Statement

The studies involving human participants were reviewed and approved by University of Minnesota, Institutional Review Board (IRB). Written informed consent to participate in this study was provided by the participants’ legal guardian/next of kin.

## Author Contributions

KQ lead the writing and data analyses in close collaboration with JT, ME, RW, C-SG and BZ. DP aided in scripting and coding of preprocessing analyses. KC wrote the introduction and contributed to the synthesis of all manuscript parts.

## Conflict of Interest

The authors declare that the research was conducted in the absence of any commercial or financial relationships that could be construed as a potential conflict of interest.

## Footnotes

^1^www.nitrc.org/projects/artifact_detect/
