# Supplementary material for: Amygdala Circuitry During Neurofeedback Training and Symptoms’ Change in Adolescents With Varying Depression
Source: Front Behav Neurosci. 2020 Jul 22;14:110. doi: 10.3389/fnbeh.2020.00110 (PMC7388863; doi:10.3389/fnbeh.2020.00110)
Supplement: Supplementary file 1 [file Presentation_1.pdf]

I. Online Steps to Warp the AMYHIPP ROI to each Individual Brain.

Generation of subject specific anatomic masks of the bilateral amygdala and hippocampus (AMYHIPP) region of interest (ROI). The single band reference functional image from a pre-feedback multiband EPI series was used as the target functional reference for the coordinate system transformation since the neurofeedback would be generated from subsequent real-time multiband EPI within the same imaging session. The high-resolution structural image of the subject (MPRAGE) was also used as a structural anatomic reference for registration to the MNI reference. An AMYHIPP mask derived via the WFU_PickAtlas tool (Maldjian, Laurienti, Kraft, & Burdette, 2003) was transformed from MNI space into subject's functional imaging space using SPM12 modules The 4-step process was as follows: 1. Alignment of the subject's functional and structural images series. 2. Segmentation and spatial normalization of the structural image to the MNI coordinate space and output of spatial normalization parameters that perform inverse deformation between coordinate spaces. 3. Warping of the ROI from MNI space to subject space using the spatial normalization parameters yielded by step 2. 4. Registration of the warped ROI to match voxel-for-voxel the specific subject's functional image space to allow for real-time masking. Masks were converted from 8bit to 16 bit for compatibility with MURFI. This last step concluded with overlapping of the ROI on both structural and functional images. **Supplemental Figure S. 1.**

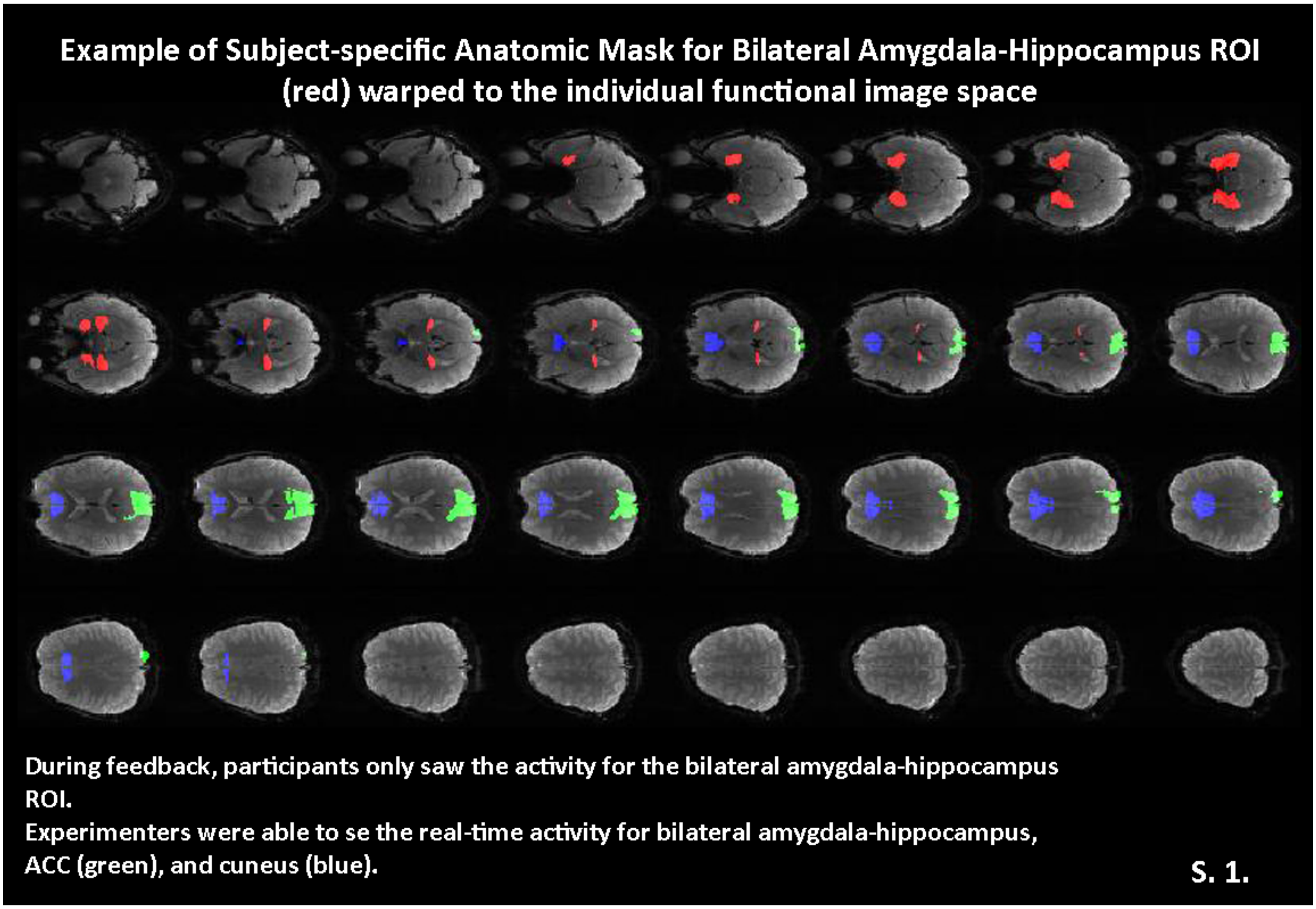

II. Memory check and happiness before versus NF. Ratings of happiness before and after the scanning as well as memory recall ratings were analyzed with a repeated measures ANOVA and a one-way ANOVA respectively. A 10-point scale rating measured successful recalling of happy memories during the ESOM_NF task, and happiness before and after NF. There were no significant differences, $F(1,48)=0.397$, $p = 0.53$, for ratings in successful recalling of happy memories between control (M=5.53) and depressed (M=5.18) groups. Additionally, analyses of ratings for happiness before and after scanning task showed that healthy control youth tended to have a higher rating in happiness overall at both times. However, the groups did not differ in their ratings before and after the scanning tasks $F(1,50)=2.91$, $p=0.09$.

III. Peak amygdala coordinates in 8 mm spheres during the ESOM_NF task.

Table S1. Peak Amygdala Coordinates (Mean ± Standard Error).

Axis	Healthy Control	Depressed
	**Left Amygdala**	
X	-23.98 ± .845	-24.233± .669
Y	-1.048 ± .782	-1.808± .619
Z	-13.798 ± 1.560	-15.250± 1.235
	**Right Amygdala**	
X	25.845± 1.153	25.617± .913
Y	-.821± .881	-.700± .697
Z	-16.810± 1.152	-15.883± .912

IV. General linear model analyses showed that during Emotion Self-Other Morph_Neurofeedback (ESOM_NF) all youth rated their affect as more positive after feedback+self-face+autobiographical memory versus count-backward+other-face blocks (i.e. feedback vs. count-backwards conditions), $F_{Emotion\ Self\text{-}Other\ Morph\ Neurofeedback\ Condition}(1, 21)=6.6$, $p<0.05$. Additionally, all youth were slower to rate their affect after feedback vs. count-backwards blocks, $F_{Emotion\ Self\text{-}Other\ Morph\ Neurofeedback\ Condition}(1, 21)=6.6$, $p<0.05$ perhaps indicating more thoughtfulness or internal mental activity after active NF blocks. There were no group effects or group by condition interactions on ratings or reaction times during the ESOM_NF task.
