# Supplementary material for: Amygdala Circuitry During Neurofeedback Training and Symptoms’ Change in Adolescents With Varying Depression
Source: Front Behav Neurosci. 2020 Jul 22;14:110. doi: 10.3389/fnbeh.2020.00110 (PMC7388863; doi:10.3389/fnbeh.2020.00110)
Supplement: Supplementary file 2 [file Data_Sheet_1.PDF]

Consensus on the Reporting and Experimental Design of clinical and cognitive-behavioural Neurofeedback studies (CRED-nf) best practices checklist 2019*

Domain	Item #	Checklist item	Reported on page #
**Pre-experiment**			
	1a	Pre-register experimental protocol and planned analyses	not clinical
	1b	Justify sample size	Page 6.
**Control groups**			
	2a	Employ control group(s) or control condition(s)	Page 7-8.
	2b	When leveraging experimental designs where a double-blind is possible, use a double-blind	NA
	2c	Blind those who rate the outcomes, and when possible, the statisticians involved	NA
	2d	Examine to what extent participants and experimenters remain blinded	NA
	2e	In clinical efficacy studies, employ a standard-of-care intervention group as a benchmark for improvement	NA
**Control measures**			
	3a	Collect data on psychosocial factors	Table 1
	3b	Report whether participants were provided with a strategy	Page 7
	3c	Report the strategies participants used	Autobiographical Memories
	3d	Report methods used for online-data processing and artifact correction	Page 8
	3e	Report condition and group effects for artifacts	
**Feedback specifications**			
	4a	Report how the online-feature extraction was defined	Page 8
	4b	Report and justify the reinforcement schedule	Page 8
	4c	Report the feedback modality and content	Page 8 and 9
	4d	Collect and report all brain activity variable(s) and/or contrasts used for feedback, as displayed to experimental participants	Page 9-10
	4e	Report the hardware and software used	Page 8
**Outcome measures**			
Brain	5a	Report neurofeedback regulation success based on the feedback signal	Quevedo 2019
	5b	Plot within-session and between-session regulation blocks of feedback variable(s), as well as pre-to-post resting baselines or contrasts	Quevedo 2019
	5c	Statistically compare the experimental condition/group to the control condition(s)/group(s) (not only each group to baseline measures)	Page 9
Behaviour	6a	Include measures of clinical or behavioural significance, defined a priori, and describe whether they were reached	Page 6
	6b	Run correlational analyses between regulation success and behavioural outcomes	Table 3
**Data storage**			
	7a	Upload all materials, analysis scripts, code, and raw data used for analyses, as well as final values, to an open access data repository, when feasible	Not feasible at this time

*Darker shaded boxes represent *Essential* checklist items; lightly shaded boxes represent *Encouraged* checklist items. If a checklist item is "not addressed" in the manuscript, enter "N" in place of the page number. We recommend using this checklist in conjunction with the CRED-nf article, which explains the motivation behind this checklist and provides details regarding many of the checklist items.
